# Different imaging patterns of PCNSL and IVL: a case report

**DOI:** 10.1186/s12883-019-1548-3

**Published:** 2019-12-03

**Authors:** Seung-Ho Jeon, Mi-Kyoung Kang, Seung Jae Lee, Byoung-Soo Shin, Hyun Goo Kang

**Affiliations:** 1 Department of Neurology, Jeonbuk National University Medical School and Hospital, Jeonju, South Korea; 2 Institute for Molecular Biology and Genetics and Department of Chemistry, Jeonbuk National University, Jeonju, South Korea; 3 Biomedical Research Institute, Jeonbuk National University, Jeonju, South Korea

**Keywords:** Central nervous system, Encephalopathy, Lymphoma

## Abstract

**Background:**

Primary central nervous system lymphoma (PCNSL) is a rare, malignant, non-Hodgkin’s lymphoma of the brain, leptomeninges, and rarely the spinal cord. PCNSL has characteristic magnetic resonance imaging (MRI) findings, and effective treatment strategies are available. It is characterized predominately by neurological symptoms, which are caused by tumor infiltration into the nervous system as well as ischemia. Chemotherapy is an effective treatment, if started prior to the ischemic damage.

**Case presentation:**

A 62-year-old male patient with PCNSL presented with altered mental status. The initial brain MRI revealed high signal intensity on the T2-weighted images (T2WIs) of the putamen area of the right basal ganglia, and the clinical symptoms improved after steroid administration. However, the symptoms were later deteriorated, we considered the possibility of autoimmune encephalitis and, consequently, conducted an immunomodulatory therapy. In a follow-up brain MRI, enlargement lesions of T2WI in basal ganglia and pons were simultaneously enhanced. Subsequently, the patient’s mental status deteriorated to a semi-coma and PCNSL was diagnosed after a surgical biopsy. Chemotherapy was started immediately; however, the patient died.

**Conclusions:**

Effective treatments are available for PCNSL and intravascular lymphoma; thus, their prognosis is generally good if they are diagnosed early. Herein, we report the case of a patient suspected with autoimmune encephalitis after brain MRI and treated with immunomodulation therapy. However, PCNSL was confirmed by a surgical biopsy. It is, therefore recommended to consider lymphoma in patients with neurological symptoms that are difficult to localize and rapidly progressive enhancing lesions showing a mass effect on brain MRI.

## Background

Primary central nervous system lymphoma (PCNSL) is a rare disease that constitutes less than 1 or 2% of all non-Hodgkin’s lymphoma cases and accounts for 3–6% of all primary brain tumors. PCNSL presents with focal neurologic signs that are associated with the mass effect [1]. Intravascular lymphoma (IVL) is an extra-nodal non-Hodgkin’s lymphoma that initially develops in the intima of small arterioles and is commonly diagnosed in the brain and skin. The incidence rate of IVL is less than one person per one million people, and it is diagnosed by the presence of malignant lymphocytes in the small arterioles [2].

It is difficult to accurately diagnose these two diseases using early-stage brain magnetic resonance imaging (MRI). Moreover, it is necessary to distinguish PCNSL from other neuroinflammatory disorders, such as malignant glioma, metastases, and demyelinating disease [3], and IVL from an ischemia-induced lesion [2]. However, effective treatments are known for these two diseases and previous studies indicate that appropriate treatment can greatly increase a remission rate of each disease. Therefore, it is particularly important to diagnose these diseases at an early stage and start treatment as soon as possible [4].

Herein, we report the case of a patient with PCNSL in whom the diagnosis was delayed because the patient did not show a sufficient response to the steroid treatment and his brain MRI did not show any typical findings of PCNSL.

## Case presentation

**Fig. 1:**
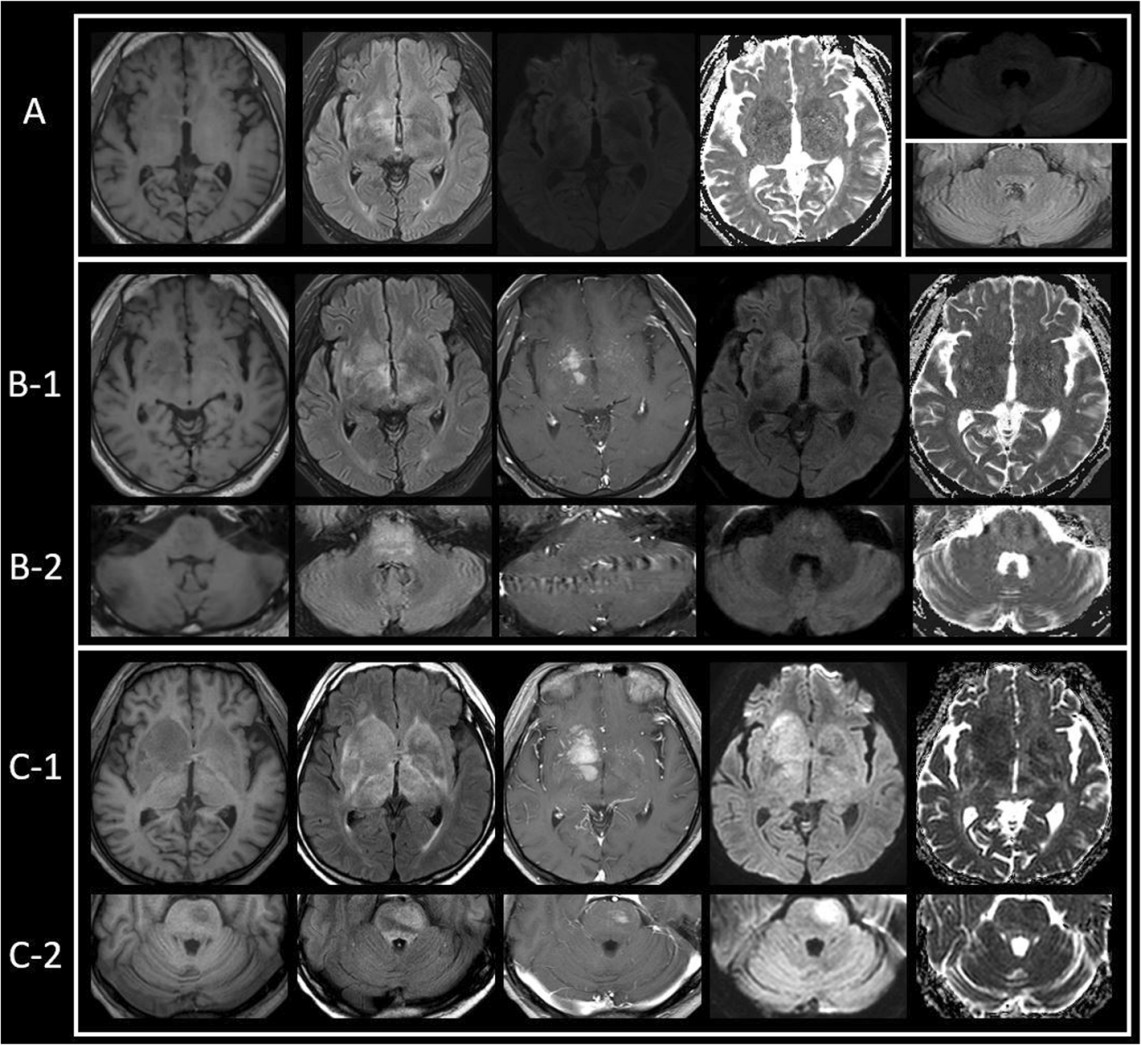
Serial brain magnetic resonance imaging (MRI) of the patient. (A) First MRI after the onset of symptoms. The right-sided basal ganglia had a mild, low signal intensity on the T1-weighted image and high signal intensity on the fluid attenuated inversion recovery (FLAIR) image. However, matching of diffusion weighted image (DWI) and apparent diffusion coefficient (ADC) was not clearly observed. The pons and cerebellar pontine angle don’t have any abnormal signal intensity on the DWI and FLAIR image. Abnormal signal intensity was not observed in FLAIR in both medial temporal lobes. (B) Follow-up MRI after symptom aggravation. Compared with first MRI, the signal intensity in the T1-weighted image was pronounced and the size of the high signal intensity on the FLAIR image increased. Furthermore, enhancement of the lesion was observed as well as matching of the DWI and ADC. (B-1) A new lesion, which was not seen in the first MRI, was observed in the pons. Low signal intensity was observed on the T1-weighted image and high signal intensity on the FLAIR image. Matching of the DWI and ADC was observed. (B-2). (C) After plasmapheresis, the patient’s condition worsened, and follow-up brain MRI was performed. The basal ganglia enhancement was increased and the edema worsened. The matching of DWI and ADC became more prominent. (C-1) Enhancement lesion was observed in the pons. (C-2)

A 62-year-old male, who was previously in good health, was admitted to our hospital due to mental status deterioration, which exacerbated five days before the admission. The patient had presented to a local clinic with dysarthria and gait ataxia 20 days before the admission. Brain MRI conducted at the clinic revealed high signal intensity lesions on the T2-weighted image (T2WI) of the right putamen, right thalamus, and bilateral hypothalamus (Fig. 1-A). The patient was administered dexamethasone 5 mg three times per day for 3 days. All neurological symptoms improved and the patient was discharged 3 days after admission. However, 20 days after discharge and 23 days after the onset of symptoms, the dysarthria exacerbated along with mental status alteration. Therefore, the patient visited the same local clinic and brain MRI was performed again, which revealed contrast enhancement and enlargement of the central area in the existing putamen lesion. Moreover, a new lesion with a strong signal was observed in the pons (Fig. 1-B). The patient was administered 1 g of methylprednisolone per day for 3 days. The gait ataxia improved but his mental status alteration persisted. As a result, the patient was transferred to our hospital 5 days after the occurrence of dysarthria and mental status alteration.

The patient had been diagnosed with diabetes mellitus 3 years prior to this visit and was taking medication. Two weeks before symptom onset, the patient had been diagnosed with early-stage gastric cancer from a biopsy, and was scheduled to undergo endoscopic mucosal resection. At the time of admission, his blood pressure was 110/50 mmHg, the heart rate was 68 bpm, the respiratory rate was 18/min, and the body temperature was 37.4 °C. The neurological examination revealed a drowsy mental status, moderate dysarthria, and dysmetria. The vocal quality was harsh and it was spastic dysarthria with low pitch. The cerebellar dysfunction test showed an overshoot pattern indicating that coordination was lacked, which had more dominant at the left side in both upper and lower limbs. However, there was no abnormal finding in the pupil reflex and cranial nerve function tests. Moreover, muscle weakness of limbs and hypesthesia were not observed, and deep tendon reflex was normal as well. However, the right Babinski reflex was dorsiflexion of the toe.

The laboratory examination showed increased white blood cell (WBC) count of 12,000 counts/μl, while the liver and renal function test and inflammatory markers, such as erythrocyte sedimentation rate (ESR, 6 mm/hr) and C-reactive protein (CRP, 0.47 mg/L), were within the normal range. The cerebrospinal fluid (CSF) revealed a slight increase in the pressure to 20 cmH_2_O, and the color was transparent. WBC count in the CSF was 14 counts/mm^3^, and the protein level was in the normal range of 41.9 mg/dl. The glucose level of CSF was 112 mg/dl, which was slightly higher than the normal range (50–75 mg/dl). In CSF cytopathology, lymphocyte-dominant chronic inflammatory cells were identified, and there were no atypical cells such as malignant cells.

Plasmapheresis was performed daily for 5 days because paraneoplastic syndrome or autoimmune encephalitis was suspected. However, the level of consciousness further deteriorated to semi-coma and spasticity was observed at both upper and lower limbs of the patient. The follow-up brain MRI revealed enlargement of the existing high signal intensity lesion of the putamen in the T2WI and linear enhancement of the central part along the arterioles (Fig. 1-C). The pons high signal intensity lesion on the T2WI was also increased, with contrast enhancement at the central area.

**Fig. 2:**
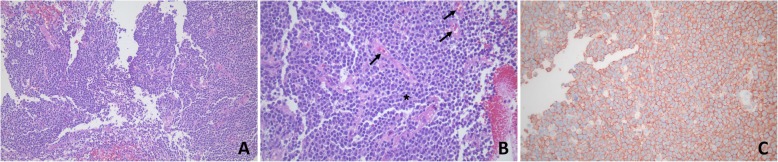
The outcome of brain biopsy. **a** Hematoxylin and eosin (H&E) stained slide (× 200) shows a diffuse large B-cell lymphoma. The neoplastic cells have a uniform cytological appearance with medium-sized to large lymphoid cells with usually oval to round vesicular nuclei containing fine chromatin. The cytoplasm is usually scant and amphophilic. **b** Hematoxylin and eosin (H&E) stained slide (× 400) shows large malignant tumor cells with prominent single central nucleoli (star). Malignant cells were not observed in the intravascular space (arrow). (C) CD20 immunostaining (× 400). It shows strong membranous and perinuclear labeling of the malignant cells for CD20

Brain biopsy was performed for an accurate diagnosis and primary diffuse large B cell central nervous system lymphoma was diagnosed, as large malignant lymphoid cells with positive CD20 immunostaining were observed and there was no specific lesion in the blood vessels (Fig. 2). Chemotherapy with R-CHOP (Rituximab-cyclophosphamide, doxorubicin, vincristine, and prednisone) was administered immediately, but the symptoms worsened and the patient died.

## Discussion and conclusions

The patient in the present case report showed neurological abnormalities that were not localized as a focal lesion. Moreover, it was difficult to explain these abnormalities using the brain MRI or to identify the cause from the physical examinations. According to the diagnostic criteria for autoimmune encephalitis by Lancet Neurology in 2016 [5], although it was not a lesion limited to the medial temporal lobe in the T2WI, progressive neurological abnormalities of subacute onset and CSF pleocytosis were observed, suggesting autoimmune encephalitis. Immunomodulation therapy was administered because autoimmune encephalitis was suspected, but with no effect. The follow-up brain MRI revealed a progressive pattern that could not be explained by a general vascular cause. Although PCNSL was diagnosed with brain biopsy, the brain MRI of the patient did persistently show a unique lesion, not typical for PCNSL. There was a partial response to the early steroid therapy; however, the symptoms reoccurred. The possibility of a neuroinflammatory disease was low as the patient did not respond to steroids. We diagnosed the patient with PCNSL based on the biopsy results and immediately started chemotherapy.

IVL, on the other hand, has clinical symptoms similar to those of other diseases, such as demyelinating disease, vasculitis, stroke, and cerebral autosomal dominant arteriopathy with subcortical infarcts and leukoencephalopathy (CADASIL). Therefore, it is exceedingly difficult to diagnose it promptly and accurately [6]. Due to the nonspecific clinical symptoms and imaging findings, more than half of the patients with IVL are diagnosed with IVL post-mortem [7].

PCNSL presents on brain MRI as a solitary, homogenous, strong contrast-enhancing parenchymal mass. Moreover, the edema observed on the T2WI correlates with the size of the enhanced contrast lesion on the T1-weighted images. These characteristics reflect the characteristics of PCNSL such as hypercellularity, high nuclear/cytoplasmic ratio, and the disruption of the blood-brain barrier. The lesion is usually located in the central hemisphere or periventricular cerebral white matter. It occurs most commonly in the frontal lobe. The brain MRI findings of IVL are multiple abnormalities on the diffusion-weighted images (DWIs) accompanied with an abnormal signal on the T2WI and contrast enhancement, which precedes the T2WI or DWI changes and persists for several weeks or several months. And various changes in the subcortical white matter or the occurrence of a new lesion are observed on the T2WI or the DWIs [8]. In our patient, high signal intensity was observed on the T2WI of the early MRI, but it did not present as an acute ischemic stroke on the DWIs and the apparent diffusion coefficient (ADC) map. In the follow-up image, a contrast-enhanced lesion and increased edema were observed on the T2WI. Moreover, in the DWI and ADC map, an acute ischemic lesion and a large-size edema compared to the size of the contrast enhancement were observed. In this case, the edema was larger than the contrast-enhanced lesion and the contrast enhancement started after a signal change was found on the T2WI and DWIs. These patterns were different from the conventional findings on images of PCNSL or IVL.

Corticosteroids have cytotoxic effects on B lymphocytes and are used as a supplementary treatment to reduce the PCNSL lesion [9]. Therefore, they can affect the biopsy findings. However, a previous study showed that the administration of corticosteroids prior to biopsy did not influence the outcome [10]. The results imply that the cytotoxicity of corticosteroids does not affect the diagnosis and the administration of corticosteroids should not be delayed for histopathological diagnosis. In this case, a corticosteroid was administered before conducting the biopsy, but it was not difficult to diagnose lymphoma histopathologically. However, the radiological finding was different from that typical of PCNSL in that the contrast enhancement occurred later than the changes in the T2WI and DWIs. It is believed that the difference was caused by the delay in contrast enhancement, which could have been induced by the cytotoxic effect of the corticosteroid, administered before taking the contrast-enhanced image, on the lymphoma cells. At the same time, it is anticipated that the cytotoxic effects could have delayed the increase in edema, and as a result, it showed an abnormal pattern. Moreover, although lymphoma cells were abundant in the brain biopsy samples, it is possible that they were not found in the intravascular biopsy samples because of the cytotoxic effect of the corticosteroid in the blood vessels. Performing a brain biopsy is a challenging task and it poses a high risk of complications and sequelae. A skin biopsy, on the other hand, is relatively simple to conduct. IVL is mainly found in the skin and central nervous system. Therefore, the skin biopsy can be a more effective diagnosis method, even before administering a corticosteroid [11].

The treatment for IVL and PCNSL is similar to that for non-Hodgkin’s lymphoma. A previous study showed that chemotherapy using the R-CHOP (Rituximab, cyclophosphamide, doxorubicin, vincristine, and prednisone) regimen significantly improved the complete remission rate and the final survival rate of patients with PCNSL [4]. As seen in this case, although PCNSL has effective treatments, the prognosis may be poor if the diagnosis is delayed. This is particularly true for IVL, which shows different radiological findings and clinical features from PCNSL [11].

We believe that it is necessary to consider the possibility of PCNSL or IVL in patients with DWI lesions different from a general ischemic stroke and non-localized neurological abnormality, or non-typical brain MRI findings, such as linear enhancement along the arterioles without a laboratory finding indicating an autoimmune disease. Since PCNSL and IVL are treatable diseases, it would increase the possibility of successful treatments and better prognosis.

## Data Availability

All data generated or analyzed during this study are included in this published article.

## Abbreviations

ADC: Apparent diffusion coefficient
CADASIL: Cerebral autosomal dominant arteriopathy with subcortical infarcts and leukoencephalopathy
CSF: Cerebrospinal fluid
DWI: Diffusion-weighted image
FDG: F18-Fluorodeoxyglucose
IVL: Intravascular lymphoma
MRI: Magnetic resonance imaging
PCNSL: Primary central nervous system lymphoma
PET: Positron emission tomography
T2WI: T2-weighted image
WBC: White blood count
